# Models and data of AMPlify: a deep learning tool for antimicrobial peptide prediction

**DOI:** 10.1186/s13104-023-06279-1

**Published:** 2023-02-02

**Authors:** Chenkai Li, René L. Warren, Inanc Birol

**Affiliations:** 1 Canada’s Michael Smith Genome Sciences Centre, BC Cancer Agency, Vancouver, BC V5Z 4S6 Canada; 2 Bioinformatics Graduate Program, University of British Columbia, Vancouver, BC V6T 1Z4 Canada; 3 Public Health Laboratory, British Columbia Centre for Disease Control, Vancouver, BC V5Z 4R4 Canada; 4 Department of Pathology and Laboratory Medicine, University of British Columbia, Vancouver, BC V6T 1Z4 Canada; 5 Department of Medical Genetics, University of British Columbia, Vancouver, BC V6H 3N1 Canada

**Keywords:** Antimicrobial peptide, Deep learning, Imbalanced classification

## Abstract

**Objectives:**

Antibiotic resistance is a rising global threat to human health and is prompting researchers to seek effective alternatives to conventional antibiotics, which include antimicrobial peptides (AMPs). Recently, we have reported AMPlify, an attentive deep learning model for predicting AMPs in databases of peptide sequences. In our tests, AMPlify outperformed the state-of-the-art. We have illustrated its use on data describing the American bullfrog (*Rana [Lithobates] catesbeiana*) genome. Here we present the model files and training/test data sets we used in that study. The original model (the balanced model) was trained on a balanced set of AMP and non-AMP sequences curated from public databases. In this data note, we additionally provide a model trained on an imbalanced set, in which non-AMP sequences far outnumber AMP sequences. We note that the balanced and imbalanced models would serve different use cases, and both would serve the research community, facilitating the discovery and development of novel AMPs.

**Data description:**

This data note provides two sets of models, as well as two AMP and four non-AMP sequence sets for training and testing the balanced and imbalanced models. Each model set includes five single sub-models that form an ensemble model. The first model set corresponds to the original model trained on a balanced training set that has been described in the original AMPlify manuscript, while the second model set was trained on an imbalanced training set.

## Objective

Antimicrobial peptides (AMPs) are gaining great interest as a potential replacement for progressively ineffective conventional antibiotics [1, 2]. In previous work, we built a deep learning model, AMPlify, with bidirectional long short-term memory (Bi-LSTM) and attention mechanisms for AMP prediction [3]. The model displays state-of-the-art performance, and has identified four novel AMPs from the bullfrog genome [4]. We have released a command-line based tool that implements AMPlify, publicly available at https://github.com/bcgsc/AMPlify.

This data note has two main objectives. First, we provide users with flexibility and convenience in accessing the data for training and testing AMPlify, or using the trained models. The original model files and data sets are easily accessible from a public repository, should users wish to embed or adapt our method into their own pipelines. Second, we provide an auxiliary model for use cases where AMPs must be predicted from imbalanced sets of peptide sequences composed of many more non-AMP sequences and few AMP sequences.

The model described in our earlier publication was trained on a balanced set containing the same number of AMP and non-AMP sequences [3]. The balanced model works well on relatively curated candidate sets (e.g. mature sequences derived from candidate AMP precursors identified by homology approaches), as applied in our previous work [3]. For other cases where the candidate sets are less curated and where AMPs comprise only a small proportion (e.g. filtering through an entire genomics and/or transcriptomics data set), keeping the number of false positives to a minimum would be essential. The imbalanced model reported here gives the flexibility to predict AMPs from larger sequence databases.

## Data description

### Training and test sets

In our study, we built balanced and imbalanced training and test sets to both train and assess our AMP prediction models. Table 1 lists two AMP and four non-AMP sequence sets that form the aforementioned training and test sets [5]. All AMP sequences were obtained from two curated databases: Antimicrobial Peptide Database [6] (APD3, http://aps.unmc.edu/AP) and Database of Anuran Defense Peptides [7] (DADP, http://split4.pmfst.hr/dadp). All non-AMP sequences were sampled from the UniProtKB/Swiss-Prot [8] (https://www.uniprot.org) database.

**Table 1:** Overview of data files/data sets

Label	Name of data file/data set	File types (file extension)	Data repository and identifier (DOI or Accession number)
Data file 1	AMPlify_balanced_model	.zip	Zenodo (10.5281/zenodo.7320306) [5]
Data file 2	AMPlify_imbalanced_model	.zip	Zenodo (10.5281/zenodo.7320306) [5]
Data file 3	AMPlify_data_note_supplementary_materials	.pdf	Zenodo (10.5281/zenodo.7320306) [5]
Data set 1	AMPlify_AMP_train_common	.fa	Zenodo (10.5281/zenodo.7320306) [5]
Data set 2	AMPlify_non_AMP_train_balanced	.fa	Zenodo (10.5281/zenodo.7320306) [5]
Data set 3	AMPlify_AMP_test_common	.fa	Zenodo (10.5281/zenodo.7320306) [5]
Data set 4	AMPlify_non_AMP_test_balanced	.fa	Zenodo (10.5281/zenodo.7320306) [5]
Data set 5	AMPlify_non_AMP_train_imbalanced	.fa	Zenodo (10.5281/zenodo.7320306) [5]
Data set 6	AMPlify_non_AMP_test_imbalanced	.fa	Zenodo (10.5281/zenodo.7320306) [5]

Data set 1 (3338 AMPs) and Data set 2 (3338 non-AMPs) in Table 1 form the balanced training set, while Data set 3 (835 AMPs) and Data set 4 (835 non-AMPs) form the balanced test set. These balanced training and test sets have been published in the original study. Non-AMP sequences were selected through keyword filtering and sampled matching the number and length distribution of the AMP sequences. Detailed method of how non-AMP sequences were sampled can be found in the Methods section of the original AMPlify manuscript [3].

Data set 1 (3338 AMPs) and Data set 5 (102,756 non-AMPs) in Table 1 form the imbalanced training set, while Data set 3 (835 AMPs) and Data Set 6 (25,689 non-AMPs) form the imbalanced test set. Unlike the balanced training and test sets, we took all the 128,445 available non-AMP sequences selected through keyword filtering as described in the original manuscript [3] to form the imbalanced training and test sets without selecting the same number of samples as the positive sets. The ratio of the number of non-AMPs versus AMPs were kept the same across the imbalanced training and test sets (~ 30.8). It is worth noting that there is no overlap between any of the training and test set pairs.

All the aforementioned data sets were uploaded to Zenodo (https://zenodo.org) for better accessibility, and included in the AMPlify Github repository (https://github.com/bcgsc/AMPlify) for convenience.

### Models

In this study, we acquired two sets of models, with each set containing five single sub-models that form the final ensemble model. Single sub-models were trained on different training subsets, and their output probabilities were averaged to form the final ensemble model. Please refer to the Methods section of the original AMPlify manuscript for ensemble method details [3]. All the models were built and saved by Keras 2.2.4 [9] with Tensorflow 1.12.0 [10] as the backend in Python 3.6.7. Note that we only saved the weights of the models to minimize the size of each file. An example of how to load the models can be found in the Python script *AMPlify.py* from our GitHub repository (https://github.com/bcgsc/AMPlify). Our models were saved in HDF5 (.h5) format, and each model set packaged into a zip file (Table 1, Data file 1 and Data file 2) [5].

The first set of models (Data file 1) was trained on the balanced training set with all settings described in the AMPlify manuscript [3]. This set of five single sub-models forms the original ensemble model of AMPlify (the balanced model) that has been published (i.e. AMPlify version 1.0), and the discovery of the four novel AMPs presented in the AMPlify manuscript used this specific model [3]. It is suitable to use for predicting putative AMPs from a relatively curated candidate set.

The second set of models (Data file 2) was trained on the imbalanced training set. Different from the training of the previous set of models that applied early stopping (i.e. the model stops training if there’s no further improvement in the next 50 epochs) [3], this set of models was trained using a fixed number of epochs considering the long training time on a huge training set. The best tuned number of epochs was 50. Furthermore, we tuned a best set of class weights (AMP: 0.8333, non-AMP: 0.1667) when calculating the loss function during training to ensure that the model would not bias too much on the majority class, while the loss calculations of the two classes were treated equally for the balanced model (in other words, with a weight ratio of 1:1). Both the optimal epoch number and class weights were tuned based on accuracy scores from five-fold cross-validation. This set of five single sub-models forms a new ensemble model of AMPlify (the imbalanced model), which finds applications in situations where the number of non-AMPs in the input sequence set is far greater than that of AMPs.

All models were uploaded to the repositories along with our data sets. These two sets of models together form the version 1.1 of AMPlify. We also provide a PDF file (Data file 3) [5] that includes a detailed analysis of the performance of our models under different input conditions, so that users can get a better understanding of which models to use.

## Limitations

There are limitations to both our models and data sets. Since the number of positive training samples (known AMPs) is limited compared with that of other typical deep learning applications, such as natural language processing or computer vision tasks, the performance of our AMP prediction models has some room for improvement. On the other hand, the negative samples (non-AMPs) we collected were mainly based on keyword filtering through the UniProtKB/Swiss-Prot [8] database, since there is little information available from public databases that reports peptide sequences without antimicrobial activities. Although we listed as many keywords as possible when doing filtering, this strategy might still leave some sequences there that do have antimicrobial activities but have never been tested or reported. We would expect the noise level in the non-AMP sets to decrease as more peptides are characterized in future studies, refining the quality of annotations.

## Data Availability

The data described in this Data note can be freely and openly accessed on https://zenodo.org under 10.5281/zenodo.7320306. Please see Table 1 and reference [5] for details and links to the data.

## Abbreviations

AMP: Antimicrobial peptide
APD: Antimicrobial Peptide Database
Bi-LSTM: Bidirectional long short-term memory
DADP: Database of Anuran Defense Peptide
